# Binding of non-canonical peptidoglycan controls *Vibrio cholerae* broad spectrum racemase activity

**DOI:** 10.1016/j.csbj.2021.01.031

**Published:** 2021-01-26

**Authors:** Akbar Espaillat, Cesar Carrasco-López, Noelia Bernardo-García, Alzoray Rojas-Altuve, Javier Klett, Antonio Morreale, Juan A. Hermoso, Felipe Cava

**Affiliations:** a Department of Molecular Biology and Laboratory for Molecular Infection Medicine Sweden, Umeå Centre for Microbial Research, Umeå University, Umeå, Sweden; b Department of Crystallography & Structural Biology, Institute of Physical-Chemistry “Rocasolano”, CSIC, Madrid 28006, Spain; c Centro de Biología Molecular Severo Ochoa, CSIC, Madrid 28049, Spain

**Keywords:** Bsr, Broad-spectrum Racemase, NCDAA, Non-Canonical D-Amino Acids, LAA, L-Amino Acids, DAA, D-Amino Acids, Glu-R, Glutamate Racemase, Ala-R, Alanine Racemase, TEV, Tobacco Etch Virus, SDS, Sodium Dodecyl Sulfate, HPLC, High Performance Liquid Chromatography, DAAO, D-Amino Acid Oxidase, FDAA, 1-fluoro-2-4-dinitrophenyl-5-L-alanine, M, N-acetyl-muramic acid, G, N-acetyl-glucosamine, m-DAP, l,d-Diaminopimelate, NCDAA, BsrV, Peptidoglycan, *Vibrio cholera*, Negative feedback loop

## Abstract

Broad-spectrum amino acid racemases (Bsrs) enable bacteria to generate non-canonical D-amino acids (NCDAAs), whose roles and impact on microbial physiology, including modulation of cell wall structure and dissolution of biofilms, are just beginning to be appreciated. Here we used a diverse array of structural, biochemical and molecular simulation studies to define and characterize how BsrV is post-translationally regulated. We discovered that contrary to *Vibrio cholerae* alanine racemase AlrV highly compacted active site, BsrV’s is broader and can be occupied by cell wall stem peptides. We found that peptidoglycan peptides modified with NCDAAs are better stabilized by BsrV’s catalytic cavity and show better inhibitory capacity than canonical muropeptides. Notably, BsrV binding and inhibition can be recapitulated by undigested peptidoglycan sacculi as it exists in the cell. Docking simulations of BsrV binding the peptidoglycan polymer generate a model where the peptide stems are perfectly accommodated and stabilized within each of the dimeŕs active sites. Taking these biochemical and structural data together, we propose that inhibition of BsrV by peptidoglycan peptides underlies a negative regulatory mechanism to avoid excessive NCDAA production. Our results collectively open the door to use “à la carte” synthetic peptides as a tool to modulate DAAs production of Bsr enzymes.

## Highlights

• Cell wall muropeptides bind *Vibrio cholerae's* broad spectrum racemase BsrV and inhibit its activity.
• BsrV activity is regulated by polymeric peptidoglycan.
• Muropeptides modified with non-canonical D-amino acids show the strongest inhibitory activity of BsrV.

## Graphical abstract

**Figure:**
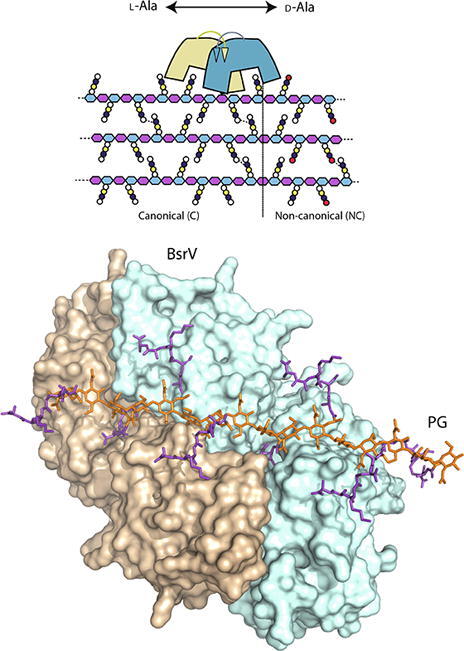

## 1 Introduction

Amino acids exist as both L and D enantiomers, being the L-form the most predominant [1]. Whist L-amino acids (LAA) are the building blocks of proteins in all kingdoms of life [2], the presence of D-amino acids (DAA) is usually linked to the existence of dedicated amino acid racemases, which are able to interconvert L to D-amino acids and vice versa [3]. The most commonly studied DAA racemases are the Ala-racemase (Ala-R) and the Glu-racemase (Glu-R) implicated in the synthesis of D-Ala and D-Glu, main components of the bacterial peptidoglycan, also called murein sacculus [4], [5]. The bacterial peptidoglycan is an indispensable net-like subcellular structure formed by groups of sugars (N-acetyl-glucosamine-N-acetyl-muramic acid) cross-linked by short peptides chains that include both LAA and DAAs [6]. The archetypical peptide stem structure is L-alanine, D-glutamic acid, a dibasic amino acid (typically *meso*-diaminopimelic acid or L-lysine), D-alanine, and D-alanine. Therefore, both cytoplasmic racemases Ala-R and the Glu-R, as well as their reaction products (DAA), are fundamental to maintain the murein sacculi structure and, thus, bacterial fitness [6].

Bacteria can have several copies of both these racemases [7]. For example, *Vibrio cholerae* presents two non-functional redundant Ala-R, one of which is primarily related to peptidoglycan biosynthesis. Interestingly, *V. cholerae* encodes an additional multispecific amino acid racemase, named BsrV for broad-spectrum racemase *Vibrio,* which produces non-canonical DAAs (NCDAAs), i.e., DAAs that are different from those usually present in the cell wall [8], [9]. BsrV is an Ala-R homolog. As Ala-R, BsrV uses pyridoxal 5-phosphate (PLP) as a cofactor and can efficiently racemize Ala. However, BsrV produces nine additional DAAs, including the non-β-branching aliphatic amino acids (Leu, Met, Ser, Cys, Gln and Asn) and the positively charged amino acids (His, Lys and Arg) [9].

In *V. cholerae,* expression of BsrV is regulated by the stress sigma factor RpoS in response to high population density and nutrient exhaustion (i.e., stationary growth phase) [8]. In this context, BsrV is expressed, and its activity drives the production and release of millimolar concentrations of NCDAAs to the extracellular media. NCDAAs can then be used as substrates by certain cell wall synthetic enzymes to induce chemical changes in the peptidoglycan composition [8], [10], [11]. It has been demonstrated that such cell wall chemical editing by NCDAA down-regulates peptidoglycan synthesis to enable cell wall adaptation to stationary phase conditions [8], [10], [11].

In addition to being regulators of peptidoglycan synthesis and integrity [8], [10], [11], NCDAA has also been reported to be involved in diverse cellular processes such as catabolism [12], biofilm formation [13], bacteria-bacteria interactions [14], microbiome biodiversity [15], modulation of host immune cells, and immune cell response [16]. As their L-enantiomeric counterparts, the physiological role of NCDAAs depends both on each particular bacteria and the chemical properties of the NCDAA produced [7], [17]. Nonetheless, high levels of DAA are usually detrimental to most bacterial species. So, it has been hypothesized that NCDAA-producing bacteria should be equipped with a regulatory mechanism to tolerate the toxic effects of these molecules [18].

In a previous study, we reported the three-dimensional structure of BsrV and defined a molecular fingerprint of conserved residues that define the family of broad-spectrum racemases [9]. Compared to Ala-R, BsrV’s capacity to accommodate amino acid substrates other than Ala capitalizes on its broader entry channel and active site. Using a BsrV-His variant, we observed that the hexahistidine affinity tag was fully stabilized in BsrV’s entry channel. Based on these data, we proposed that BsrV might interact with the cell wall muropeptides. Using *in vitro* biochemical and structural analyses, we demonstrated that peptidoglycan peptide moieties bind and inhibit BsrV activity. Interestingly, edited muropeptides containing NCDAAs (produced by BsrV) showed stronger binding and inhibitory properties compared to those ending in D-Ala (canonical muropeptides), suggesting that BsrV activity is controlled via a negative feedback loop by the degree of NCDAA cell wall editing.

## 2 Materials and methods

### 2.1 Microbiology

All *V. cholerae* strains used in this study were derived from the sequenced El Tor clinical isolate N16961 [23] and were grown on Luria-broth (LB) medium with 1% NaCl. Strains, plasmids and primers, growth conditions and mutant bacterial strains, and standard molecular biology techniques are described below.

### 2.2 Protein expression and purification

The *V. cholerae* and *A. hydrophila* genes encoding BsrV, Bsr_Ah_, Alr_Ah_, and BsrV His-tagged less were cloned on pET28b (Novagen) for expression in *E. coli* BL21 (*DE3*) cells [24]. Expression was induced (at OD600 = 0.4) with 1 mM IPTG for 3 h. Cell pellets were resuspended in 50 mM Tris HCl pH 7.2, 150 mM NaCl, 10% glycerol, and Complete Protease Inhibitor Cocktail Tablets (Roche), and lysed with 3 passes through a French press were purified from cleared lysates (30 min, 50000 rpm) on Ni-NTA agarose columns (Qiagen) and eluted with a discontinuous imidazole gradient. Pure proteins were visualized by SDS-PAGE electrophoretic protein separation [25]. BsrV was purified from its His-tagged derivative (see Table S2), which presents a Tobacco etch virus (TEV) protease cleavage site preceding the His-tag were cloned in pET28b (Novagen). TEV protease (Sigma) digestion was performed at 30 °C for 6 h, in 25 mM Tris-HCl, pH 8.0, 150–500 mM NaCl, 14 mM β-mercaptoethanol.

### 2.3 Peptidoglycan analysis

Peptidoglycan sacculi were prepared by pelleting 500 mL of bacterial cells. Cell pellets were resuspended into a small volume of medium and slowly dropped into an equal volume of boiling 10% (w/v) SDS. The sacculi were ultracentrifuged for 15 min at 100,000 rpm (TLA110 Beckman rotor; OptimaTM Max ultracentrifuge Beckman), and the pellets were washed 3 times by repeated cycles of centrifugation and resuspension in water. The pellet from the last washing was resuspended in 300 µL of 50 mM sodium phosphate buffer pH 4.5, and subjected to overnight digestion with 30 µg/mL muramidase (cellosyl, Hoescht) at 37 °C. Muramidase digestion was stopped by incubation on a boiling water bath (5 min) [26]. The supernatants were mixed with 150 µL 0.5 M sodium borate pH 9.5 and subjected to reduction of muramic acid residues into muramitol by sodium borohydride (10 mg/mL final concentration, 30 min at RT) treatment. Samples were adjusted to pH 3.5 with phosphoric acid. HPLC analyses of muropeptides were performed on an Aeris peptide reverse-phase column (250 × 4.6 mm; 3.6 μm particle size) (Phenomenex, USA) and detected by Abs. (204 nm), using a linear gradient of Phosphate buffer/methanol. Muropeptides were quantified from their integrated areas using concentration standards as described [26]. The identity of individual D-Met-and D-Arg-muropeptides was established by MALDI-TOF (Autoflex, Bruker Daltonics)

### 2.4 Interaction between protein and muramidase-digested sacculi

1 mg of His-tagged proteins (BsrV, AlrV and AmpC) were immobilized in 1 mL of Ni-NTA resin (Qiagen) in sodium phosphate buffer 100 mM pH 7.5. Each protein was subjected to incubation with exact equal amounts of muramidase treated sacculi (120 µg of muropeptides, 1 mL) for 10 min at 25 °C after which the eluted fraction was collected in a gravitational column. Then, the column was washed with 1 mL sodium phosphate buffer 100 mM pH 7.5, also collecting this fraction. Both fractions were quantified by HPLC analysis and the areas obtained were rested to the original input of muropeptides assayed, giving the percentage of muropeptides interacting with the proteins.

### 2.5 Racemase activity assays

For activity assays, *in vitro* LAA and DAAs were characterized with Marfey’s (FDAA)-derivatization in HPLC and DAAO assay (D-amino acid oxidase) performed as described [9]. The product from a racemization reaction was derivatized with L-FDAA (1-fluoro-2-4-dinitrophenyl-5-L-alanine amide, Marfey’s reagent, Thermo Scientific). First, an equal volume of NaHCO_3_ 0.5 M was added to the racemization reaction; then, 6 µL of this reaction was reacted with FDAA (10 mg/mL in acetone) at 80 °C for 3 min. The reaction was stopped with HCl 2 N and the samples were filtered. The products were separated with a linear gradient of triethylamine phosphate/acetonitrile in HPLC with an Aeris peptide column (250 × 4.6 mm; 3.6 μm particle size) (Phenomenex, USA) and detected at Abs.340 nm. To determine the inhibition effect of the sacculus in BsrV's activity, 35 µg of sacculi were incubated for 5 min at 37 ˚C with BsrV and 4 mM of L-Ala in Tris-HCl 50 mM pH 8. The product was revealed with DAAO [21]. DAAO reaction was determined by coupling 10 µL of the extract into 150 µL of a reaction containing: sodium phosphate buffer 100 mM pH 7, Trigonopsis variabilis D-amino acid oxidase (DAAO) (Komarova et al., 2012) 3.6 U/ml, horseradish peroxidase 1 U/mL, o-phenylenediamine (OPD) 2 mg/mL and FAD 3 mg/ml. This two-step assay permits the quantification of H_2_O_2_ (DAAO is able to produce α-ketoacid, NH_3_ and H_2_O_2_ from DAA). Peroxidase reduces H_2_O_2,_ releasing free O_2_ that reacts with OPD, leading to the production of 2,3–25 diaminophenazine. The reaction was incubated for 2 h at 37 °C and inactivated with HCl 3 M, giving a colorimetric product that can be measured at 492 nm. To determine the inhibition effect of muropeptides in BsrV's activity, 0.1 mM of M4 (GlcNAc-MurNAc-Ala-Glu-DAP (Diaminopimelate)-Ala), M3M (GlcNAc-MurNAc-Ala-Glu-DAP-Met), M3R (GlcNAc-MurNAc-Ala-Glu-DAP-Arg) and D-cycloserine were incubated with BsrV and 4 mM of L-Ala for 5 min at 37 ˚C (1/40 relation) in Bicarbonate buffer 50 mM pH 9. In the case of tripeptide and D-Ala-D-Ala, equal concentration (amino acid, tripeptide/dipeptide) was utilized. The product was revealed with Marfey's- reagent as described above.

### 2.6 Structural determination

Crystallization of BsrV tagless was performed as previously described for the His-tagged proteins [9]. Briefly, a high-throughput NanoDrop ExtY robot (Innovadyne Technologies Inc.), the commercial Qiagen screens The JCSG + Suite and The PACT Suite and the Hampton Research screens Index, Crystal Screen and Crystal Screen 2 were used to get crystals by the sitting-drop vapor-diffusion method. Best crystals were obtained with 0.1 M Bis-Tris propane pH 7.5, 0.2 M Sodium Iodide, and 24% (p/v) of PEG 3350. X-ray data collection was performed on the X06SA beamline at the SLS synchrotron-radiation facility in Villigen, Switzerland. Data sets were collected using a PILATUS 6 M detector, and were processed using XDS [27] and scaled using SCALA [28] from the CCP4 suite [29]. The structure was solved by the molecular replacement method with MOLREP [30] from the CCP4 suite using the His-tagged version of BsrV (PDB code 4BEU) as initial model. Refinement was performed with PHENIX [31] and modeling with Coot [32]. The stereochemistry of the models was verified using MolProbity [33]. A summary of the data collection and refinement statistics is given in Table 1.

**Table 1:** Data collection and refinement statistics.

	BsrV-Tagless
Wavelength (Å)	1.0
Resolution range (Å)	51.2–1.52 (1.57–1.52)
Space group	P 2_1_ 2_1_ 2_1_
Unit cell	
*a, b, c* (Å)	54.03, 82.09, 160.21
α, β, γ (°)	90, 90, 90
Total reflections	17,752,204
Unique reflections	111,214
Multiplicity	7.2 (8.2)
Completeness (%)	99.9 (99.9)
Mean I/ σ(I)	14 (4.2)
R-merge	0.092 (0.562)
R-pim	0.036 (0.205)
CC1/2	0.981 (0.819)
Reflections used in refinement	111,036 (10943)
R-work/ R-free	0.1799 /0.1984
Number of non-hydrogen atoms	6732
macromolecules	5965
ligands	44
solvent	723
Protein residues	776
RMS bonds (Å)	0.018
RMS angles (°)	1.63
Ramachandran favored (%)	97.40
Ramachandran allowed (%)	2.60
Ramachandran outliers (%)	0.00
Average B-factor (Å2)	20.68
macromolecules	19.12
ligands	26.61
solvent	33.21
PDB code	7AGZ

Statistics for the highest-resolution shell are shown in parentheses.

### 2.7 Docking and molecular dynamic simulations

Standard MD simulations were run using the CUDA version of the sander module in the AMBER 12 suite of programs [34]. The resulting systems were simulated under the same conditions up to a total time of 10 ns during which system coordinates were collected every 2 ps for further structural and energetic analysis. Binding energy evaluation and decomposition were achieved through MM-ISMSA scoring function [35].

### 2.8 Statistical analysis

The program GraphPad PRISM® Software (Inc., San Diego CA, www.graphpad.com) has been used for all statistical analyses. To determine the significance of the data displayed in Fig. 3, the *t*-test (unpaired) has been performed. P-values smaller than 0,05 were considered statistically significant, with the following ranking: p < 0,05(*); p < 0,001(***).

### 2.9 Data availability

The atomic coordinates and structure factors for BsrV His-Tagged less (PDB 7AGZ) have been deposited in the Protein Data Bank, Research Collaboratory for Structural Bioinformatics, Rutgers University, New Brunswick, New Jersey, USA (http://www.rcsb.org/). The rest of the data are contained within this manuscript.

## 3 Results

### 3.1 BsrV C-terminal His-tag interacts with the enzyme catalytic channel

**Fig. 1:**
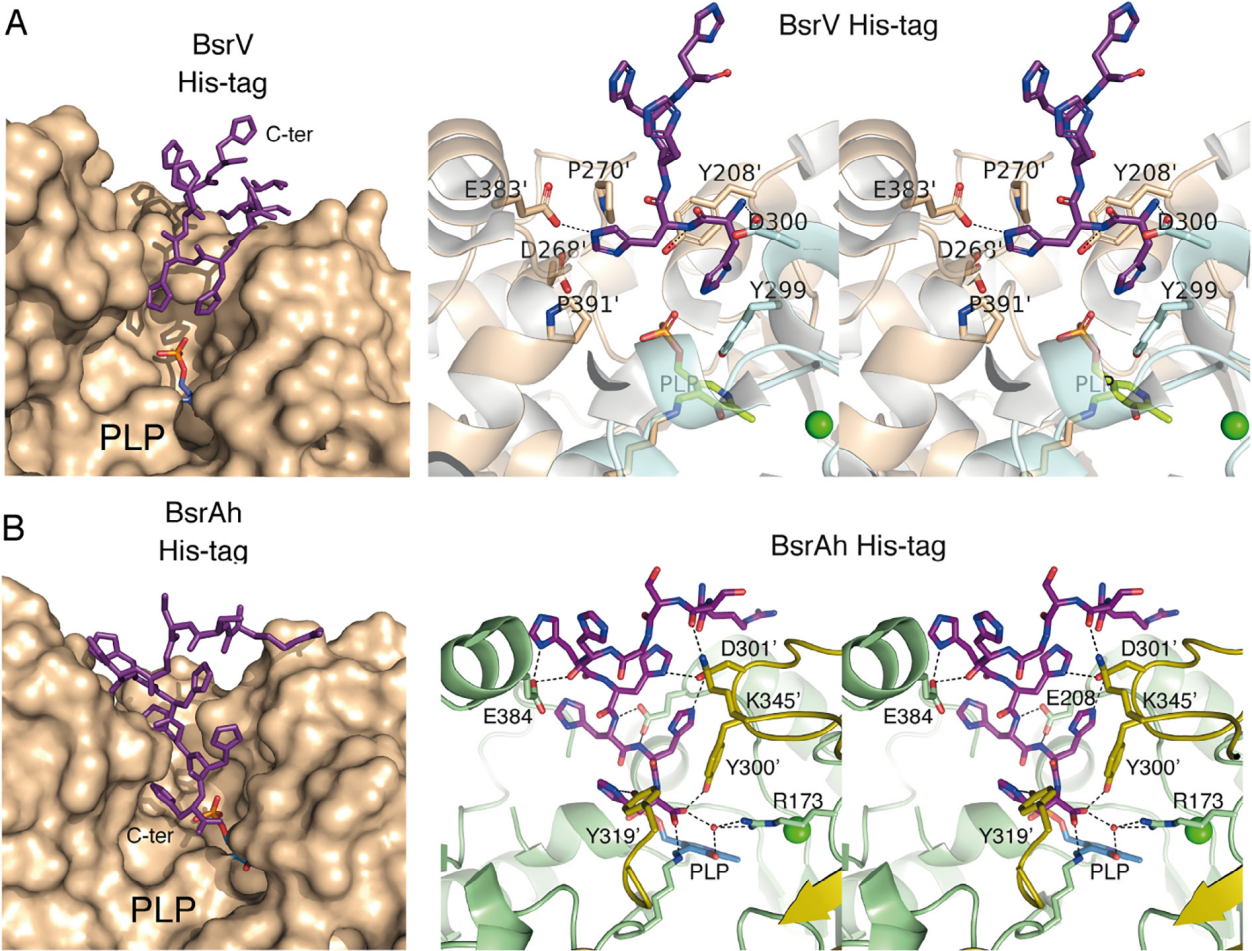
Muropeptide recognition by BsrV. Crystal structure of (A) BsrV and (B) Bsr_Ah_ active site entry. Left, the molecular surface of one monomer is colored in brown (the surface for the partner is omitted for clarity). The C-terminal His-tag (purple sticks) of a crystal partner enters into the BsrV (A) and Bsr_Ah_ (B) active sites. Righ, stereoview showing the polar interactions (dotted lines) between the His-tag and the Bsr active site. Catalytic PLP is represented in sticks and labeled. Cl^-^ ion represented as a green sphere. (For interpretation of the references to colour in this figure legend, the reader is referred to the web version of this article.)

The crystal structures of His-tagged constructs of BsrV and the broad-spectrum racemase (Bsr) from *Aeromonas hydrophila* (Bsr_Ah_) have been reported earlier [9]. Both enzymes showed a remarkable facility to crystallize [9]. After exhaustive structural analysis of BsrV and Bsr_Ah_ crystals, we realized that the C-terminal His-tag added to the racemases for purification purposes was tethering the dimers (Fig. S1). This effect was caused by the interaction of the C-terminal His-tags belonging to one dimer with the active site of adjacent dimers and thus increasing crystal contacts. Strikingly, multiple interprotein interactions were observed between Bsr’s His-tags and several residues of its catalytic channel (Fig. 1). The interactions were particularly numerous for Bsr_Ah_, whose His-tag extended through the entry site almost reaching the PLP in the catalytic site (Fig. 1, Fig. S1). In order to validate this, a His-tag less BsrV protein was crystallized and its structure was solved at atomic resolution (1.52 Å, Table 1). As expected, superimposition of the His-Tag and His-tag less BsrV revealed their strong similarity (RMSD: 0.350 Å), which is in concordance with their mirroring biochemical performance (Fig. S2) [8]. Besides minor structural changes (N207, Y208, Y246 and D300) resulting in a small decrease of around 1 Å in the entry site's aperture, no significant differences were observed in the conformation of the catalytic site (Fig. S2). The fact that Bsrs display an unusually large active site [9] capable of binding oligopeptides (His-tag) together with their periplasmic localization made us hypothesize that the stem peptides of the peptidoglycan (muropeptides) might be a more physiological interacting partner of BsrV.

### 3.2 BsrV binds to cell wall muropeptides

**Fig. 2:**
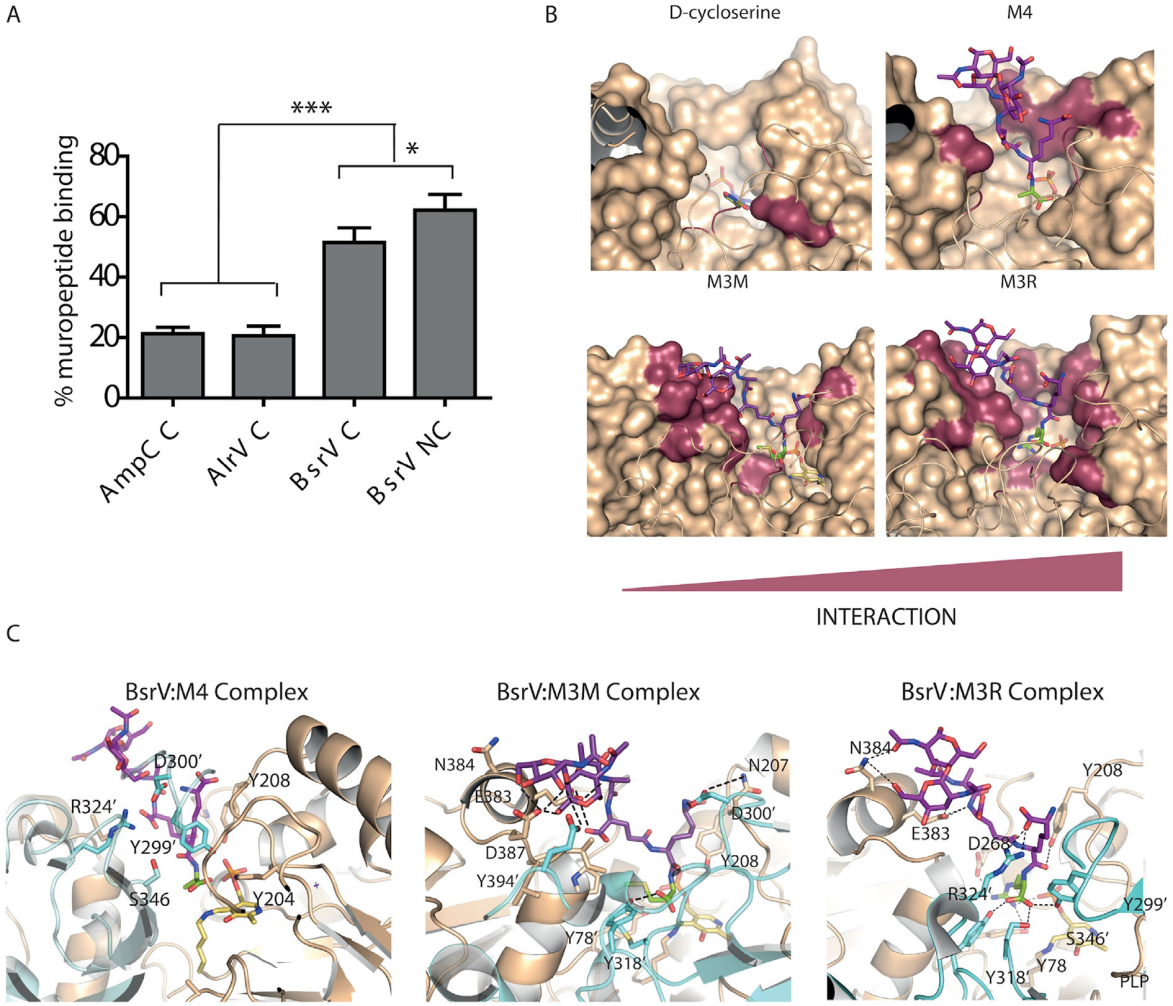
BsrV muropeptide interaction and inhibition. (A) Muropeptide relative retention by interaction with three independent proteins (AmpC (negative control), AlrV and BsrV) bound to Ni-NTA resin. Retained muropeptides are quantified by HPLC and represented as % relative to initial muropeptide load (100%). Canonical peptidoglycan (labeled C) corresponds to Δ*bsrV* digested peptidoglycan, whose terminal amino acid at the tetrapeptide moiety is always D-Ala. Non-canonical peptidoglycan (labeled NC) corresponds to digested peptidoglycan from *V. cholerae* wt cells grown on exogenous D-Met and D-Arg (9% of tetrapeptide monomers ending in D-Met and D-Arg). Results are means ± SD of triplicates from one experiment. pvalues: [AlrV(C)vsBsrV(C)] = 0.0008; [AmpC(C)vsBsrV(C)] = 0.0006; AlrV(C)vsAmpC(C)] = 0.740 4; [BsrV(C)vsBsrV(NC)] = 0.0302. (B) Docking models of D-cycloserine, M4 (disaccharide tetrapeptide), M3M (M4 with a D-Met substitution in the fourth position) and M3R (M4 with a D-Arg substitution in the fourth position) onto the BsrV active site. Molecular surface of the BsrV active site (colored in brown) is represented for one monomer (the dimeric partner is drawn in ribbons for clarity reasons). Substrates are drawn as magenta sticks—the number of interactions increases from the left (D-cycloserine) to the right (M3R). BsrV residues directly interacting with the substrates are colored in dark red onto the molecular surface. (C) Detailed view of the docking model of canonical M4 and non-canonical M3M and M3R muropeptides at BsrV active site. Models are based on the crystal structure of His-tag in BsrV and Bsr_Ah_ active sites and after molecular dynamic simulations. (For interpretation of the references to colour in this figure legend, the reader is referred to the web version of this article.)

To test this hypothesis, we compared the capacity of BsrV and AlrV (the Ala-R from *V. cholerae*) to bind muropeptides. We isolated muropeptides from *V. cholerae ΔbsrV* strain given that digestion of the peptidoglycan of this mutant renders a homogenous pool of canonical muropeptides, does not present any NCDAA-modified muropeptides (Fig. S3). BsrV retained 50–60% of *V. cholerae* isolated canonical muropeptides compared to AlrV, which retained ~ 25%. This binding appeared to be nonspecific since similar retention was observed using a control protein (AmpC-His) that does not bind peptidoglycan (Fig. 2A). Remarkably BsrV muropeptide binding increased to a 60–75% when challenged with D-Arg/D-Met muropeptides (Fig. 2A, Fig. S3), suggesting a certain specificity of BsrV for NCDAA-edited peptidoglycan.

To assess the potential fitting of muropeptides bound to BsrV’s active site, we generated docking models for several muropeptides using as template the conformation exhibited by the His-tag that is stabilized within BsrV’s active site in our crystal structure. We then performed molecular dynamic (MD) simulations of BsrV and Alr_Ec_ catalytic pockets occupied by canonical (disaccharide-tetrapeptide, GlcNAc-MurNAc-Ala-Glu-DAP-Ala; M4) and non-canonical (disaccharide-tetrapeptide with terminal d-Met or d-Arg instead of D-Ala; M3M or M3R, respectively) muropeptides (Fig. S3 and movies S1, S2 and S3). These analyses suggested that muropeptides can interact with BsrV’s catalytic channel in a manner analogous to that observed in crystal structures of the His-tagged BsrV and Bsr_Ah._ These docked complexes show many putative strong polar and hydrophobic stacking interactions between residues from the BsrV active site and all the peptide stem residues. It is noteworthy that the sugar rings (NAG, NAM) of the muropeptides can also establish polar interactions with the loops of BsrV that shape the entry of its active site cavity (Fig. 2BC). In contrast, docking/MD analyses suggested that muropeptides would encounter numerous steric clashes along the Alr_Ah_ active cavity, including the entry site, rendering this interaction very unlikely (Fig. S3). MD simulations also suggest that non-canonical muropeptides (M3M and M3R) bind to the active site of BsrV more strongly than canonical muropeptides establishing numerous hydrogen bonds and strong salt bridges (Fig. 2C), resulting in a more stable conformation of M3M and M3R in BsrV active site than M4 (movies S1, S2 and S3). This is also consistent with the results from the binding assays (Fig. 2BC, Fig. S3) and with previously reported BsrV’s selective racemization of some non-canonical substrates (e.g., Met and Arg residues over Ala) [8].

### 3.3 BsrV is inhibited by NCDAA-modified muropeptides

**Fig. 3:**
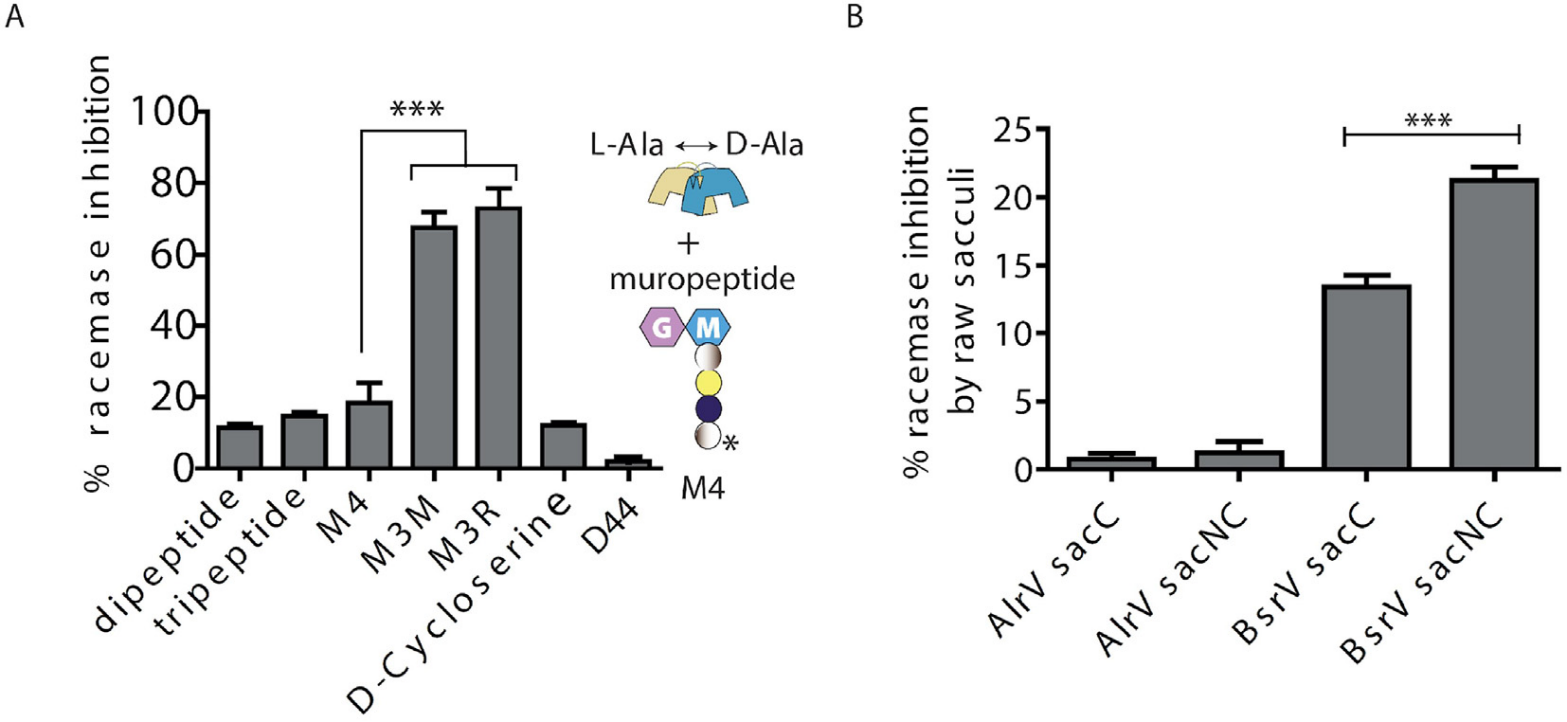
Peptidoglycan inhibits BsrV’s activity. (A) Inhibitory effect of muropeptides on BsrV activity. *In vitro* BsrV racemase assays with L-Ala as substrate in the presence of (see schematics): M4 (disaccharide tetrapeptide), M3M and M3R; the asterisk represents D-Met and D-Arg substitutions in the fourth position. Dipeptide (D-Ala-D-Ala), tripeptide (L-Ala-D-Glu-D-Lys), D44 (cross-linked M4 dimer) and D-cycloserine were also used as control. % of inhibition was calculated by subtracting these data from a control reaction with no inhibitor added, the reduction of *D*-Ala produced in the assay at a single time point. P values: [dipeptide vs tripeptide] = 0.0181; [tripeptidevsM4] = 0.0027; [M4vsM3M] = 0.0003; [M4vsM3R] < 0.0003; [M3MvsM3R] = 0.2552; [M3RvsD-cycloserine] < 0.0001. (B) Inhibitory effect of peptidoglycan sacculi on BsrV activity. *In vitro* BsrV racemase assays with L-Ala as substrate in the presence of undigested sacculi (see schematics). Sugar rings are represented by hexagons and peptides are depicted. Murein sacculi (sac) only displaying canonical muropeptides (C) or with 9% non-canonical modifications (NC). pvalue: [BsrVsacCvsBsrV sacNC] = 0.0005.

Since amino acids in muropeptides do not exhibit free amino groups linked to chiral carbons, bound peptides seemed likely to function as non-racemizable competitive inhibitors. To test this possibility, we performed *in vitro* assays of BsrV capability to racemize l-Ala in the presence or absence of different muropeptides (Fig. 3A). All monomer muropeptides assayed caused a reduction (from ~20% to 65%) in d-Ala production compared to control reactions without muropeptide. In addition, non-canonical muropeptides show a higher degree of inhibition (~65% reduction in d-Ala production) than the canonical M4 (~20% reduction), while d-cycloserine and short peptides (dipeptide and tripeptide), in general, inhibit the least (Fig. 3A). This result is likely due to the reduced number of potential interactions the small peptides can form compared to longer peptides, differentially affecting their stabilization within the active site cavity (Fig. 2B). Also, cross-linked monomers (D44) did not cause any detectable inhibition suggesting that linear peptides are needed to compete for the active site entry. Collectively, these analyses suggest that the production of NCDAAs modified muropeptides as result of BsrVs racemization of LAA might also modulate its activity *in vivo*. To further explore this possibility, we assessed whether undigested *V. cholerae* sacculi also exhibits inhibitory properties (Fig. 3B). When BsrV is incubated with canonical and NCDAA modified sacculi, we observed a significant reduction in BsrV dependent d-Ala production in the presence of NCDAAs-free sacculi (Δ*bsrV* peptidoglycan), which further decreased when sacculi containing NCDAA (9% of total muropeptides) were used instead (Fig. S4) [10]. In contrast, AlrV’s activity was not reduced by the presence of any type of sacculi, consistent with results from the docking modeling and affinity assays (Fig. 3B).

### 3.4 BsrV binds polymeric peptidoglycan

**Fig. 4:**
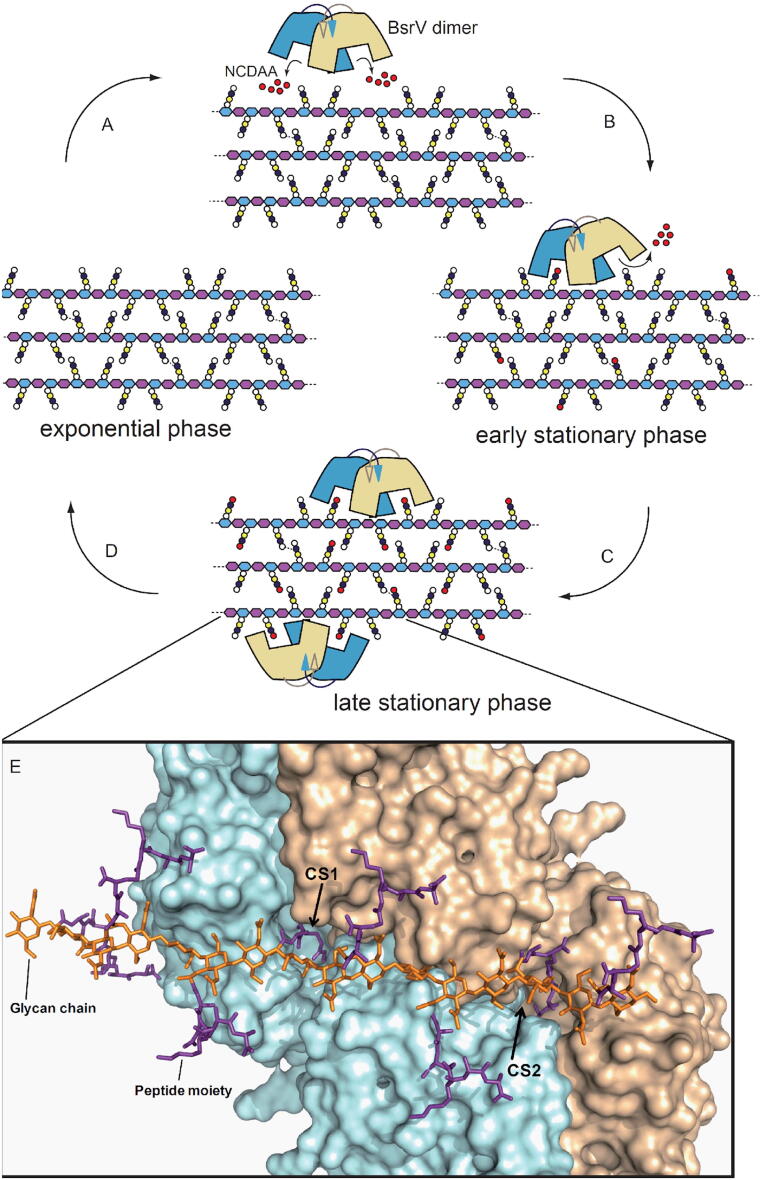
Proposed model of BsrV regulation by NCDAA in peptidoglycan. (A) In exponential growth phase, *V. cholerae* does not express BsrV and consequently, its peptidoglycan is composed only of canonical muropeptides. In the transition to stationary phase, *V. cholerae* expresses BsrV, an RpoS-dependent, periplasmic, multispecific amino acid racemase (8). BsrV produces high (millimolar) concentrations of NCDAA that accumulate in the periplasmic space and also pass to the extracellular media (10). (B) NCDAA are incorporated into peptidoglycan in stationary phase cells.peptidoglycancontaining such modifications is a more potent inhibitor of BsrV than is unmodified peptidoglycan; thus, a negative feedback loop is generated to control BsrV’s activity. (C) Ultimately, high levels of peptidoglycan modification may turn off the majority of BsrV, thereby preventing hyper modification and excessive accumulation of free NCDAA. A detail of Fig. 4 is shown. CS1 and CS2: catalytic sites. (D) BsrV expression shuts down when growth resumes, preventing further production and incorporation into peptidoglycan of NCDAA. (E) Molecular docking of the NMR-peptidoglycan structure (19) with BsrV dimer. Peptidoglycan is drawn in sticks with glycan chains colored in orange and peptide stems colored in magenta. BsrV active sites interact with the peptide moieties of the sacculus. (For interpretation of the references to colour in this figure legend, the reader is referred to the web version of this article.)

Molecular models based on NMR studies suggest that peptidoglycan forms a right-handed helical structure with the peptide stems projecting out at 120° intervals [19]. To better understand the interaction between peptidoglycan and BsrV, we ran docking simulations of peptidoglycan fragments with the BsrV molecule (Fig. 4). Notably, the distance between the active sites of a BsrV dimer and their relative rotation fit well with the peptidoglycan fragment structure reported by Meroueh et al (Fig. 4) [19]. In fact, in this model, the peptide stems from the peptidoglycan fragment are perfectly accommodated and stabilized within each of the active sites of the dimer. This precise molecular fit between the BsrV structure and the two stem peptides radiating from the same strand (separated by one helix turn lends) additionally enforce the idea that BsrV activity may be regulated by its binding to macromolecular peptidoglycan. Furthermore, our data suggest that the extent of such regulation will vary according to the NCDAAs content of the peptidoglycan, and thus inhibition will be maximal during the stationary phase, when NCDAAs incorporation into the cell wall is completed [8], [10], [11].

## 4 Discussion

We observed that BsrV can bind to muropeptides and intact peptidoglycan, particularly those containing NCDAAs. Our modeling shows that the two active sites of a BsrV dimer can be simultaneously occupied with the peptide moieties of a single peptidoglycan -strand separated by one turn. Analyses of enzyme activity, coupled with our modeling assays, suggest that muropeptides can occupy BsrV’s (but not AlrV’s) catalytic site and thereby serve as competitive inhibitors. Thus, our findings raise the possibility that the production of NCDAAs by BsrV and related periplasmic broad-spectrum racemases is down-regulated when peptidoglycan contains sufficient levels of NCDAA. Such downregulation might reflect global levels of peptidoglycan modification in the cell. Alternatively, it might also function to fine-tune the spatial allocation of NCDAAs by promoting their equal distribution throughout peptidoglycan.

Negative feedback control of BsrV activity by non-canonically modified-peptidoglycan seems reasonable given that NCDAAs reduce peptidoglycan synthesis [8], [10], [11] and that excessive concentration of NCDAAs can be lethal [18]. According to our model, the synthesis of BsrV will be produced on early stationary phase conditions in a RpoS dependent manner [8]. Following the enzyme’s translocation to the periplasm, production of NCDAAs from the corresponding l-forms ensues (Fig. 4). Since NCDAA peptidoglycan incorporation appears to be constrained to active murein biosynthetic sites [20], local concentrations of NCDAA modified muropeptides are likely to become very high, promoting their binding to and inhibition of BsrV. Inactivation of BsrV by NCDAA-modified muropeptides reduces local production of NCDAA, establishing a negative feedback loop in which the products of BsrV (Fig. 4), NCDAAs, once incorporated into peptidoglycan function as competitive inhibitors of BsrV, preventing over-production of NCDAAs that might ultimately be deleterious [18]. Moreover, in addition to the effect on *V. cholerae*, fine-tunning BsrV’s activity may also have implications on the physiology of nearby organisms as NCDAAs are known to impact a number of distinct cellular processes such as catabolism [12], biofilm formation [13], bacteria-bacteria interactions [14], microbiome biodiversity [15], modulation of host immune cells, and immune cell response [16].

The ability of BsrV to interact with peptides introduces a number of intriguing additional possibilities for the regulation of broad-spectrum racemases. For example, short linear non-ribosomal peptides (NRP), such as some secreted peptides involved in bacterial communication [21], might also interact with BsrV, either as regulators or as substrates. Given the impact of NCDAA on a variety of cellular processes [14], [15], [22], bacteria may have evolved diverse ways to control their production and to regulate its spatiotemporal allocation.

Collectively, our results open the door to use “à la carte” synthetic peptides as a tool to modulate DAAs production of Bsr enzymes. So, the effect of the DAAs in bacterial fitness and biotechnology could be modulated by the usage of diverse peptides that, in turn, would control Bsr activity.

## Declaration of competing interest

The authors declare that they have no known competing financial interests or personal relationships that could have appeared to influence the work reported in this paper.
