# Supplementary material for: Binding of non-canonical peptidoglycan controls Vibrio cholerae broad spectrum racemase activity
Source: Comput Struct Biotechnol J. 2021 Jan 26;19:1119–26. doi: 10.1016/j.csbj.2021.01.031 (PMC7890096; doi:10.1016/j.csbj.2021.01.031)

**Figure S1. His-tag packing on BsrV structure.** Molecular surface for the physiological BsrV dimer is represented with each chain colored differently (cyan and light brown). In the crystal, the C-terminal His-tags (drawn as magenta sticks) of the BsrV dimer enter into active sites of the closest BsrV chain enchaining dimers.

**Figure S2. Three-dimensional structure of His-tag less BsrV dimer.** Superimposition of the crystal structures His-tagged BsrV (light brown) and BsrV (blue). (A) detail of the C-terminal region in each protein, (B) Active-site entry (molecular surface to visualize the differences in the width for each structure, main changes highlighted by arrows) and (C) Zoomed view of the active-site entry and the catalytic site. Relevant residues are drawn as capped sticks and labeled.

**Figure S3. Muropeptide analysis of peptidoglycan for *in vitro* AlrV and BsrV reactions.**  (A) HPLC profile of muropeptides of *V. cholerae* N16961 wt (red) and Δ*bsrV* mutant (blue) in the sacculi samples used as inhibitors in BsrV racemase *in vitro* reactions (see material and methods). (B) Schematic representation and quantification of the tetrapeptides monomers presented in the samples. Relative molar abundance of muropeptides was calculated from the areas of the corresponding peaks as described previously (26). M: N-acetyl-muramic acid, G: N-acetyl-glucosamine, m-DAP: l,d-Diaminopimelate.

**Figure S4. Alr_Ec_ steric clashes and BsrV molecular simulations.** (A) BsrV:M4 complex superimposed onto Alr_Ec_ structure to show clashes. At the top, protein atoms from Alr_Ec_ structure are represented as spheres. At the bottom, residues from Alr_Ec_ structure are represented as sticks. M4 is represented as purple sticks. (B) Plot showing binding energies between BsrV and muropeptide with D-Ala (M4), D-Met (M3M) and D-Arg (M3R) attached. (C) Plot showing the RMSD (root-mean squared deviations) fluctuations for the final amino acids (D-Ala, D-Met and D-Arg) attached to the muropeptide during 10 ns MD simulations. Fluctuations in M4 complex are due to the gaining and losing of hydrogen bonds between D-Ala and His-A204, Arg-A173, LLP-A74 and Tyr-A208. Also, the fluctuations observed in the case of D-Arg complex are due to the balance between the very strong hydrogen bonds formed between the phosphate group of LLP-A74 and the Asp-A268. (F) Table showing the principal interactions (below -1 kcal/mol) formed in the three complexes.

**Video S1. Molecular dynamic simulation for M4 and BsrV interaction.**

**Video S2. Molecular dynamic simulation for M3M and BsrV interaction.**

**Video S3. Molecular dynamic simulation for M3R and BsrV interaction.**

**Supplementary Table S1. Plasmids used in this study.**

| PLASMID | DESCRIPTION | REFERENCE |
| --- | --- | --- |
| pET28b (+) | Kan^r^. lacI. Expression of genes in E. coli, dependent on T7 phage RNA polymerase. | Novagen |
| pET28b BsrV | Kan^r^. BsrV WT overexpression with 6His C-terminal fusion. (NotI/EcoRI). DNA fragment (V. cholerae’s locus VC1312) amplified with FCP43 and FCP56 primers. | (9) |
| pET28b BsrAh | Kan^r^. BsrAh overexpression. 6His C-terminal fusion. (NcoI/HindIII). DNA fragment (A. hydrofila’s locus AHA2607) amplified with FCP345 and FCP388 primers. | (8) |
| pET28b AlrAh | Kan^r^. AlrAh overexpression with 6His C-terminal fusion. (NdeI/EcoRI). DNA fragment (A. hydrofila’s locus AHA1015) amplified with FCP108 and FCP109 primers | (8) |
| pET28B  BsrV.tev.his | Kan^r^. BsrV overexpression with 6His C-terminal fusion after a Tobacco etch virus (TEV) protease cleavage site (NotI/EcoRI). DNA fragment (V. cholerae’s locus VC1312) amplified with FCP43 and FCP140 primers. | (8) |

**Supplementary Table S2. Primers used in this study.**

| NAME | SEQUENCE (5´→3´) |
| --- | --- |
| FCP43 | aaagcggccgctttcacgtagaaacgtgggttactggttcccc |
| FCP56 | aaaccatggagcagccgcttctcagtcgcaagaag |
| FCP108 | aaagaattccagtgtcttcttgtgcatg |
| FCP109 | aaatctagagcccagcagatcaccatgc |
| FCP140 | aaagcggccgcttagtgatgatgatgatgatggccgctgctggattggaagtacaggttctctttcacgtagaaacgtgggttactggttcccc |
| FCP345 | aaaccatggatgcacaagaagacactgctggccacgctg |
| FCP388 | ttagcgcttgatcttcttggggttggtgtag |

**Figure S.1**


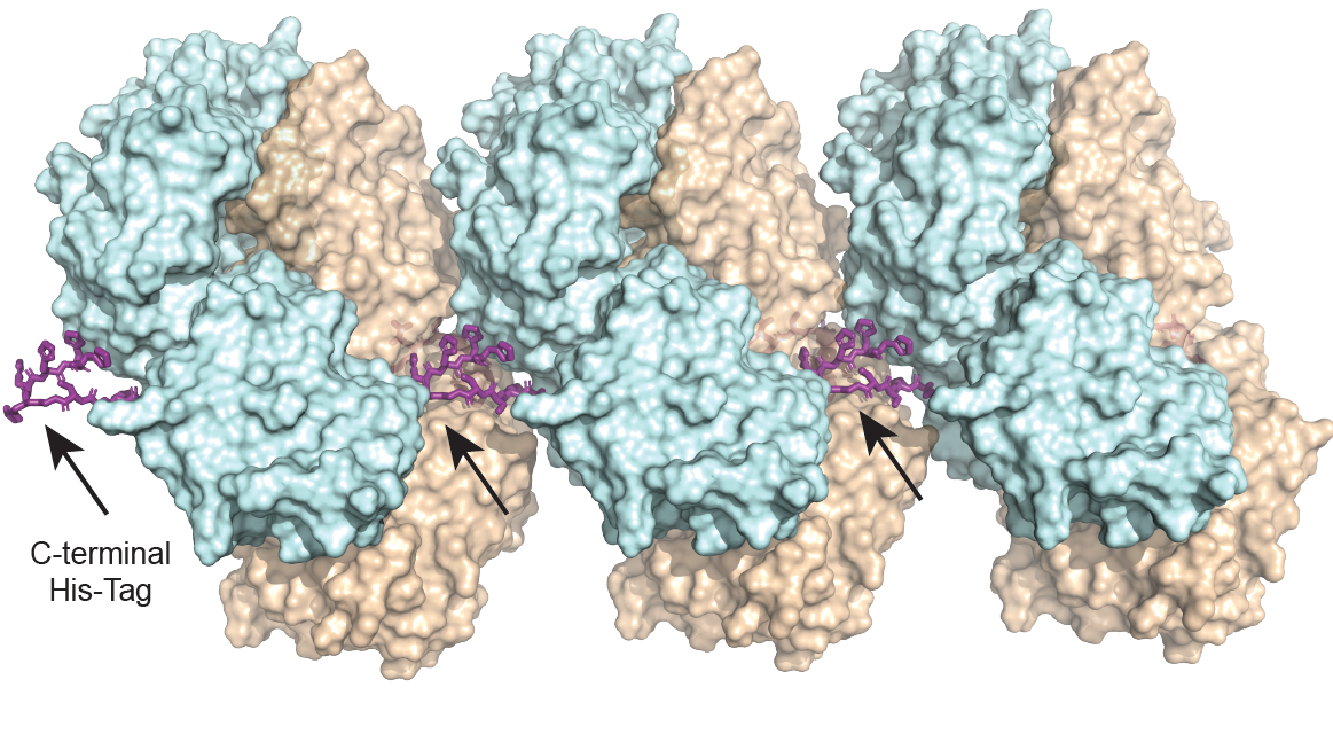


**Figure Sup.2**


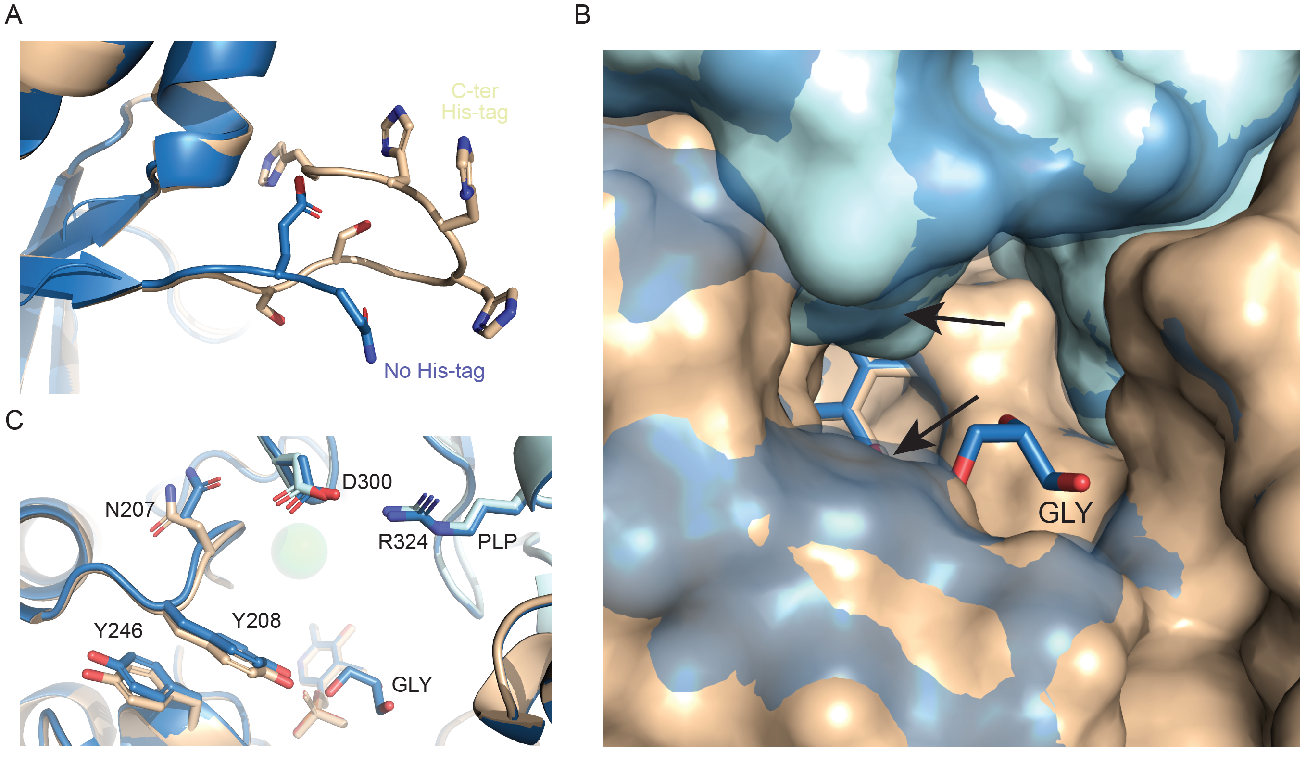


**Figure Sup.3**


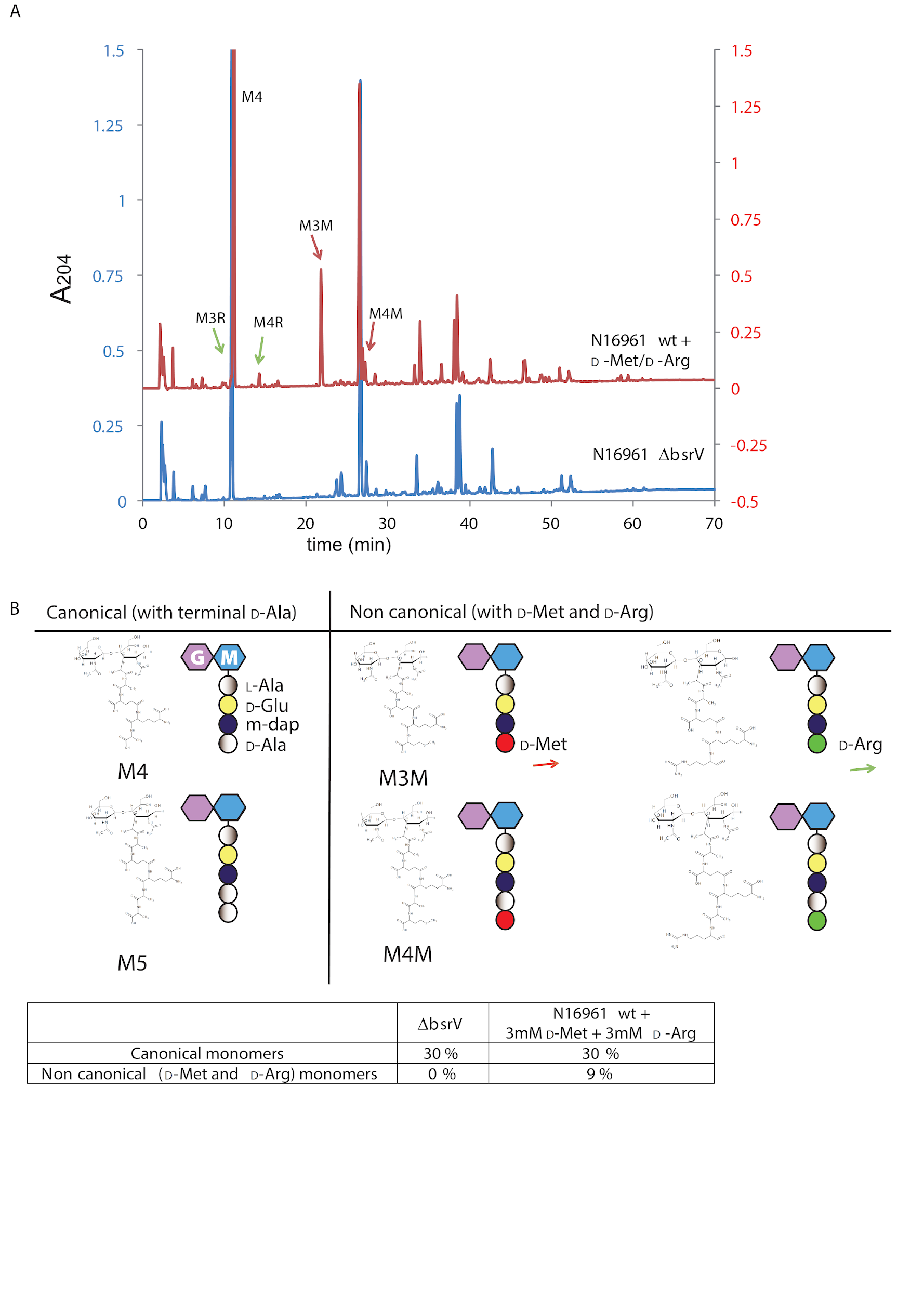


**Figure Sup.4**


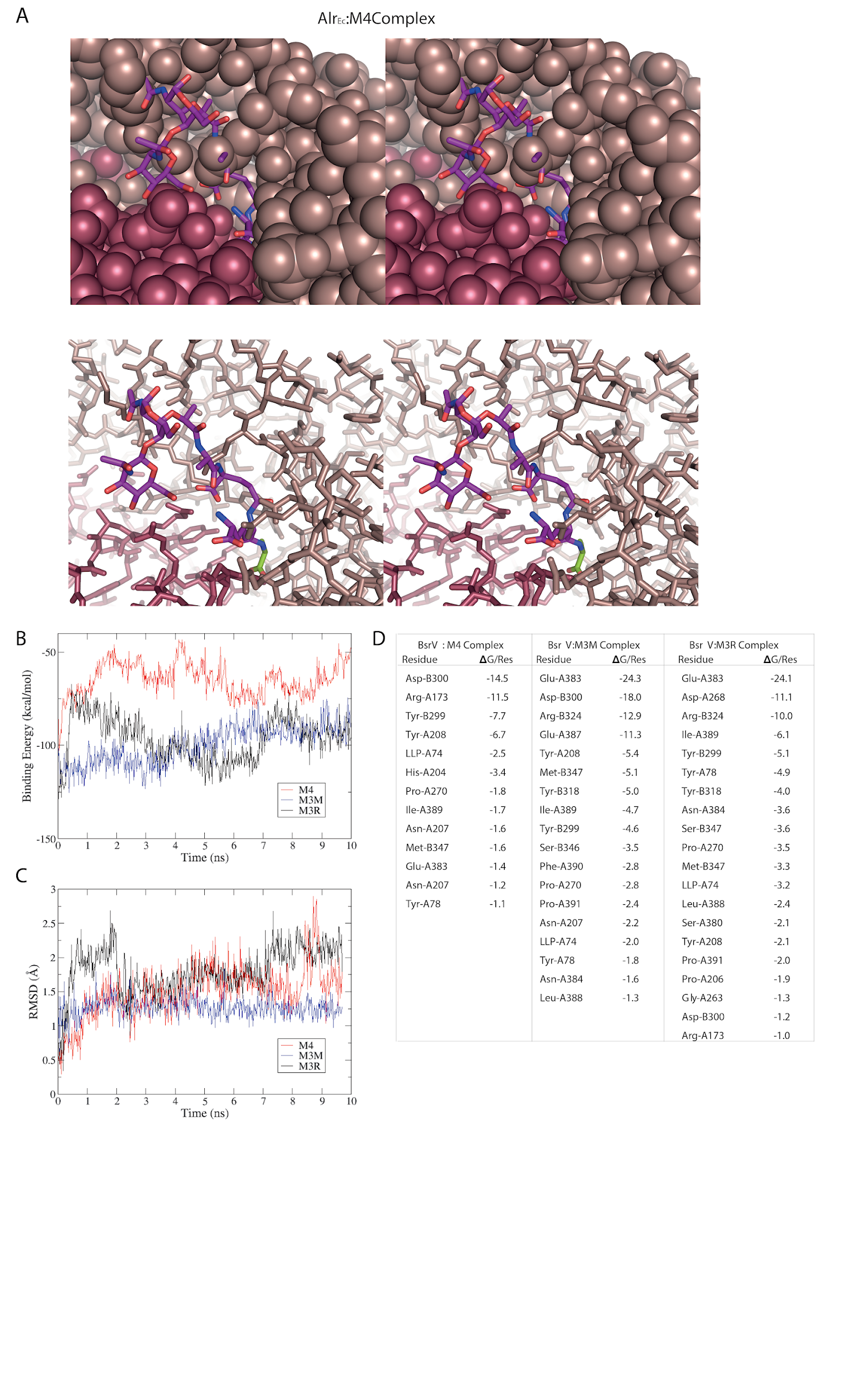

Supplement: Supplementary data 1 [file mmc1.docx]
